# In Vitro Susceptibility of* Mycobacterium ulcerans* Isolates to Selected Antimicrobials

**DOI:** 10.1155/2017/5180984

**Published:** 2017-03-14

**Authors:** Enid Owusu, Mercy J. Newman, Kwesi K. Addo, Phyllis Addo

**Affiliations:** ^1^Department of Medical Laboratory Sciences, School of Biomedical and Allied Health Sciences, University of Ghana, Accra, Ghana; ^2^Department of Medical Microbiology, School of Biomedical and Allied Health Sciences, University of Ghana, Accra, Ghana; ^3^Noguchi Memorial Institute for Medical Research, University of Ghana, Legon, Ghana

## Abstract

*Background.* The current definitive treatment of Buruli ulcer with antibiotics makes the issue of antimicrobial drug resistance an unavoidable one. This is as a result of drug misuse by health personnel and patients' noncompliance to treatment regimen. Monitoring of these factors and screening for new effective antimicrobials are crucial to effective management of Buruli ulcer disease. This study therefore investigated the inhibitory activity of some antibiotics against isolates of* Mycobacterium ulcerans*.* Methods.* Activity of eight antibiotics was tested against twelve* M. ulcerans* isolates (2 reference strains and 10 clinical isolates). The anti-*M. ulcerans* activities were determined by the agar dilution method and the minimum inhibitory concentrations (MICs) were determined by the agar proportion method.* Results.* All antimicrobials investigated had activity against* M. ulcerans* isolates tested. The MICs ranged from 0.16 *μ*g/mL to 2.5 *μ*g/mL. Azithromycin recorded the highest inhibitory activity at a mean MIC of 0.39 *μ*g/mL, whilst clofazimine a second-line antileprosy drug, recorded the lowest at a mean MIC of 2.19 *μ*g/mL. Among the four antituberculosis drugs, rifampicin had the highest activity with a mean MIC of 0.81 *μ*g/mL.* Conclusion.* Azithromycin could be considered as a lucrative alternative to existing treatment methods for inhibiting* M. ulcerans* in Ghana.

## 1. Introduction

*Mycobacterium ulcerans* is the leading cause for Buruli ulcer disease (BUD), the third most widespread mycobacteriosis after tuberculosis and leprosy [1]. The disease has been reported in over thirty countries worldwide, including Ghana [2, 3], where occurrence rates of up to 22% have been reported in certain areas [4]. The disease has been recognized by World Health Organization (WHO) as an important emerging infectious disease of public health concern [5]. BUD, a skin condition, is characterized by preulcerative and ulcerative lesions [6]. The observed clinical presentations include nodules, plaques, papules, oedemas, and ulcers. These presentations have been observed to be the cytotoxic effect of the toxin mycolactone produced by the growing* MU* in the host [6, 7].

Hitherto, the treatment of Buruli ulcer disease relied on surgical excision and repair [8]. A study by Etuaful et al. in Ghana showed that the oral administration of rifampicin and intramuscular administration of streptomycin were effective at the preulcerative and the early ulcerative stages [9]. Currently, Buruli ulcer is managed on a regimen of streptomycin and rifampicin for an eight-week period as recommended by the WHO [9, 10]. This was a shift from an initial wide surgical excision of the infected tissue, followed by skin grafting [11, 12]. In severe cases, patients are treated with antibiotics in addition to surgery. Other treatment options include topical applications such as phenytoin [13], hyperbaric oxygen, and heat [14]. Meanwhile, disease recurrence of up to 16% has been documented in Ghana [15].

Similar to most infectious diseases, the administration of antibiotics is usually recommended after laboratory confirmation of the condition. This is challenging, as the disease usually occurs in rural endemic communities with limited access to healthcare facilities [16, 17]. Even in areas where the facilities are available, polymerase chain reaction (PCR), the most sensitive technique for disease confirmation, is mostly not performed. The implication is possible misdiagnosis leading to misuse of antibiotics, an occurrence that can lead to the generation of mutant strains. Furthermore, the intramuscular administration of streptomycin can also lead to patient's noncompliance. The use of streptomycin may be inconveniencing particularly in paediatrics, since they have been identified to form a greater percentage of BUD patients [12].

Several in vitro studies have been conducted on the susceptibility of* Mycobacterium ulcerans* to antimicrobials, leading to in vivo studies and clinical trials [18–20]. However, the performance of more in vitro investigations on antimicrobials at various locations is crucial. Such investigations may bring out new antimicrobial agents useful for the management of Buruli ulcer disease in addition to what is already available. This study therefore investigated the in vitro activity of eight antimicrobials against isolates of* M. ulcerans*.

## 2. Methods

### 2.1. Study Site and Collection of Bacterial Isolates

The study was carried out at the Animal Experimental Department of the Noguchi Memorial Institute for Medical Research (NMIMR) in Ghana, after obtaining approval from Ethical and Protocol Review Committee of NMIMR and the Ghana Health Service (GHS), [Study # 040/09-10]. Two* M. ulcerans* reference strains (ATCC 970321, Ghana D19F9, and ATCC 990826, Benin D27D14) kindly donated from the laboratories of Professor Francois Portaels, Belgium, and ten clinical* M. ulcerans* isolates from Animal Experimentation and Bacteriology Departments of NMIMR, Ghana, were used for the study. The 10 isolates comprised six (6) from Amasaman and four (4) from Nsawam, both communities in Ghana. Nsawam is a town in south Ghana and is the capital of the Akuapim South Municipal District, a district in the Eastern Region of Ghana. Amasaman on the other hand is a small town and the capital of Ga West District of the Greater Accra region, located about 20 kilometers west of Accra, the capital city of Ghana [21]. A Community Health Centre is situated in the Amasaman community that cares for all Buruli ulcer patients in the Ga District and its surrounding communities [21]. Swab specimens having exudates from lesions were collected from the 10 different BU patients from Amasaman and Nsawam communities and cultured for 8–12 weeks at 32°C on Lowenstein-Jensen (L-J) medium. Growth characteristic* M. ulcerans* colonies were noticed after the incubation period [16].* M. ulcerans* colonies were confirmed by conventional laboratory methods [17], by subjecting the cultures with growth to Ziehl-Neelsen staining and a confirmatory IS2404 PCR [17]. Briefly, for the Ziehl-Neelsen staining procedure, smears were prepared from the cultures with growth and allowed to dry, after which they were heat fixed and flooded with carbol fuchsin stain. After application of heat beneath the slide until steam appeared, the slide was then left for 5 minutes while applying heat intermittently. Excess carbol fuchsin that resulted after the flooding of the slide with the stain was washed off with tap water and the excess water was drained off. Decolorization was then done by covering the slide with 20% H_2_SO_4_ and left to stand for 2–5 minutes. The slide was subsequently counterstained with 3% methylene blue and left for 2 minutes after which it was washed with plenty of water, drained, and left to dry. The stained slide was then observed with Olympus BX40 light microscope (New York Microscope Co., USA) under oil immersion. The isolates from these two towns (Nsawam and Amasaman) in addition to the reference strains were tested against eight selected antimicrobial agents, namely, amikacin, azithromycin, dapsone, ciprofloxacin, clofazimine, ofloxacin, rifampicin, and streptomycin. All the antibiotics were obtained from Fluka Biochemika (St. Louis, MO) through World Health Organization.

### 2.2. Determination of Anti-*M. ulcerans* Activities

After authentication of the MU isolates by IS2404 PCR, the anti-*M. ulcerans* activities were determined by the agar dilution method by incorporating 100 *μ*g/mL stock solutions prepared by diluting 1 mg of antibiotic in 10 mL of appropriate diluents. This was incorporated into Lowenstein-Jensen medium at a dilution ratio of 1 : 20 to obtain a final concentration of 5 *μ*g/mL.* M. ulcerans* suspensions were prepared by adjusting turbidity to MacFarland's standard solution 1. This was prepared in sterile phosphate buffered saline and matching with the standard solution equivalent to a concentration of 1 mg/mL of bacilli.

The anti-*M. ulcerans* activities of the antimicrobials were determined using the agar dilution method [22]. Briefly, labelled media test tubes containing antimicrobial-incorporated and antimicrobial-free Lowenstein-Jensen media were inoculated with 100 *μ*L of the test isolates. The tubes were gently rolled to ensure even distribution of inoculum on surface of L-J medium. The tests were done in triplicate. They were incubated at 32°C for 8 weeks under microaerophilic conditions [23]. An antimicrobial-incorporated L-J medium that had no colonies growing after 8 weeks on all the test triplicates was categorized as having anti-*M. ulcerans* activity, in vitro.

### 2.3. Determination of Minimum Inhibitory Concentrations (MICs)

The MICs of the antimicrobials were determined by Canetti's agar proportion method, modified, and used by Thangaraj et al. [19]. Briefly, the antimicrobials with anti-*M. ulcerans* activities were incorporated into L-J media at two-fold serial dilutions (1–8 dilutions). This was distributed into screw-capped tubes filled with required volumes and concentrations of antimicrobials before inspissation. The final test concentrations ranged between 0.04 *μ*g/mL and 5 *μ*g/mL in L-J medium.

Triplicates of each dilution of the antimicrobial-incorporated and antimicrobial-free media were inoculated with 100 *μ*L of the prepared inoculum containing various isolates (Ghana and Benin reference strains and the 10 clinical isolates from Amasaman and Nsawam). Noninoculated triplicates of antimicrobial-incorporated media and antimicrobial-free media were incubated alongside the inoculated ones to serve as drug and L-J media controls, respectively. All tubes were incubated at 32°C for eight weeks under microaerophilic conditions. The tubes were read weekly by observing for growth. The lowest concentration of antimicrobial that completely inhibited growth (zero colonies) was taken as the MIC.

### 2.4. Statistical Analyses

Data obtained was stored in Microsoft Excel and the analysis was conducted by the Statistical Package for the Social Sciences (SPSS), version 12.0.1. Data was summarized by determining the means, median, minimum, and maximum values of the MICs of the antibiotics. Specific determinations included differences in the MICs of the antibiotics and differences in the susceptibilities of the* M. ulcerans* isolates to the antibiotics.

## 3. Results

All twelve* M. ulcerans* isolates were susceptible to all eight antimicrobials tested. This shows that all antimicrobials had an anti-*M. ulcerans* activity. Generally, the MICs ranged between 0.16 *μ*g/mL and 2.5 *μ*g/mL, with azithromycin and rifampicin showing the highest inhibitory activity of 0.16 *μ*g/mL (Table 1). For azithromycin, the MIC of 0.16 *μ*g/mL was observed in four of the isolates (one reference and three clinical isolates). All of those isolates that were inhibited by azithromycin MIC of 0.16 *μ*g/mL were from the Amasaman community (Table 1). Meanwhile, the MIC of 0.16 *μ*g/mL for rifampicin was observed in two of the isolates, also all from Amasaman community. The isolates (A9 and A13) that were inhibited by MIC of 0.16 *μ*g/mL for rifampicin were also inhibited by the same MIC value (0.16 *μ*g/mL) for azithromycin. Clofazimine had the lowest inhibitory activity of 2.5 *μ*g/mL for nine out of twelve isolates tested (Table 1).

The best mean MIC was recorded by azithromycin (0.39 *μ*g/mL), followed by rifampicin (0.81 *μ*g/mL). Meanwhile, clofazimine performed very poorly with a mean MIC of 2.19 *μ*g/mL (Table 2). The MIC ranges for the individual antibiotics are the following: Az (0.16–0.63), Rif (0.16–1.25), Dap (0.31–1.25), Str (0.63–1.25), Amk (0.63–2.50), Cip (0.63–1.25), Ofl (0.63–2.50), and Clo (1.25–2.50) (Table 2).

## 4. Discussions

From this study, all twelve* M. ulcerans* isolates were susceptible to azithromycin, rifampicin, streptomycin, amikacin, ciprofloxacin, ofloxacin, dapsone, and clofazimine at a screening dilution of 5 *μ*g/mL. The MICs obtained for eight antimicrobials against isolates of* M. ulcerans* ranged between 0.16 *μ*g/mL and 2.5 *μ*g/mL. Azithromycin exhibited the highest inhibitory activity of 0.16 *μ*g/mL against four of the isolates while clofazimine had the lowest MIC of 2.5 *μ*g/mL against nine isolates out of the twelve tested. This observation supports outcomes of studies that have indicated that azithromycin has some level of antimycobacterial activity in vitro and in vivo [24, 25].

Between the two antileprosy drugs included in this study, dapsone had better activity than clofazimine, even though clofazimine is a well-known second-line antileprosy drug [26]. In a study by Espey et al., dapsone was administered in combination with rifampicin and it reduced the size of BU lesions impressively [27]. The fluoroquinolones, ciprofloxacin and ofloxacin, exhibited activities slightly better than clofazimine, with results comparable to results by Thangaraj et al., who also studied Ghanaian isolates [19]. However, fluoroquinolones in general are not recommended for pregnant women and children because of a possible negative impact on articular cartilage [12].

Among the four known anti-TB drugs investigated in this study (rifampicin, streptomycin, amikacin, and ciprofloxacin), the highest activity was registered by rifampicin at 0.81 *μ*g/mL, followed by streptomycin at 1.10 *μ*g/mL. The minimum inhibitory concentrations of the eight antibiotics were also in consonance with values obtained by other investigators [19, 28, 29]. This observation only confirms WHO's recommendation for the use of rifampicin combination with streptomycin as the provisional drugs for the management of Buruli ulcer [10].

Although Buruli ulcer affects all age groups, children have been identified to represent the majority of patients [12]. Therefore, it is important to assure compliance despite effects such as nausea, stomach pain, and diarrhea that patients may associate with a particular drug [12]. Streptomycin, although recommended, has a lot of limitations in terms of its mode of administration. The mode of administration makes its use in paediatrics discouraging. Rifampicin on the other hand also has the characteristics of coloring of body fluids [30].

Azithromycin has added properties such as its oral administration, rapid absorption from the gastrointestinal tract, wide distribution, its ability to penetrate and concentration in host tissue, and an efficient excretion rate [29]. In addition, it has a high peak serum concentration and a high therapeutic index. These properties of azithromycin would make it an important therapeutic agent for the disease, after its activity in vivo has been established [29]. Therefore, azithromycin would provide a more user-friendly alternative. The in vivo activity of azithromycin in combination with amikacin against* M. ulcerans* infection has been studied by Bentoucha et al. [31]. In a study conducted by Addo et al., grasscutters experimentally infected with* M. ulcerans* developed lesions that resolved and recurred after a period. The pathognomonic indicators were similar to those shown in humans [32]. This corroborated rates of recrudescence reported on the disease and the possibility of paying less attention to a seemingly resolved BU lesion. Recurrence is an important reality in BU management and antimicrobials such as azithromycin can play a very important role, particularly in the preulcerative and postsurgical periods in BU disease due to their stability and their penetrative capacity [33–35]. Additionally, multidrug regimens containing azithromycin may help control secondary bacterial infections sometimes seen in BU patients. Even though all the* M. ulcerans* isolates exhibited variable susceptibility to all the antimicrobials tested, the Nsawam isolates showed relatively lower susceptibility to the antimicrobials as compared to the Amasaman isolates, as well as the control strains. The ranges were however narrow. Exposures to antimicrobials are most likely to create decreased susceptibility as observed in some of the isolates. These are only observations in susceptibility patterns exhibited by the* M. ulcerans* isolates used in this study.

## 5. Conclusions

Azithromycin has been identified as an effective drug for controlling* M. ulcerans* isolates in Ghana. This study therefore elucidates the need to seriously and continuously conduct more susceptibility studies involving antimicrobials against isolates of* M. ulcerans*, particularly synergistic investigation of antimicrobials. This is against the backdrop that multidrug therapy tends to be more effective in antimycobacterial therapy.

## Figures and Tables

**Table 1:** Minimum inhibitory concentration (*μ*g/mL) of the antimicrobials.

Antimicrobials	MIC of *M. ulcerans* isolates (in *μ*g/mL)											
	GH	BN	A5	A9	A13	A61	A68	A69	N1	N2	N5	N8
Az	0.16	0.63	0.63	0.16	0.16	0.31	0.31	0.16	0.63	0.63	0.31	0.63
Rif	1.25	0.63	0.63	0.16	0.16	1.25	0.63	0.63	1.25	0.63	1.25	1.25
Dap	0.31	0.63	1.25	1.25	0.63	1.25	1.25	0.63	0.31	1.25	1.25	1.25
Str	0.63	1.25	0.63	1.25	1.25	1.25	1.25	1.25	1.25	0.63	1.25	1.25
Amk	0.63	1.25	0.63	2.50	1.25	2.50	2.5	1.25	0.63	1.25	0.63	1.25
Cip	1.25	1.25	1.25	1.25	0.63	1.25	1.25	0.63	1.25	1.25	1.25	1.25
Ofl	1.25	0.63	1.25	1.25	0.63	1.25	2.50	2.50	1.25	2.5	1.25	2.50
Clo	2.50	2.50	2.50	1.25	1.25	1.25	2.50	2.50	2.50	2.50	2.50	2.50

BN: Benin, GH: Ghana, A: Amasaman, N: Nsawam, Az: azithromycin, Rif: rifampicin, Dap: dapsone, Str: streptomycin, Amk: amikacin, Cip: ciprofloxacin, Ofl: ofloxacin, and Clo: clofazimine. GH and BN are lab strains.

**Table 2:** Mean, minimum, and maximum values of MIC (*μ*g/mL).

Antimicrobials	Minimum value of MIC	Maximum value of MIC	Mean MIC
Az	0.16	0.63	0.39
Rif	0.16	1.25	0.81
Dap	0.31	1.25	0.94
Str	0.63	1.25	1.10
Amk	0.63	2.50	1.36
Cip	0.63	1.25	1.15
Ofl	0.63	2.50	1.26
Clo	1.25	2.50	2.19

MIC: minimum inhibitory concentration, Az: azithromycin, Rif: rifampicin, Dap: dapsone, Str: streptomycin, Amk: amikacin, Cip: ciprofloxacin, Ofl: ofloxacin, and Clo: clofazimine.

## Abbreviations

BU: Buruli ulcer
BUD: Buruli ulcer disease
*M. ulcerans*: * Mycobacterium ulcerans*
NMIMR: Noguchi Memorial Institute for Medical Research
WHO: World Health Organization
L-J: Lowenstein-Jensen
Rif: Rifampicin
Str: Streptomycin
MTB: * Mycobacterium tuberculosis*
TB: Tuberculosis
MIC: Minimum inhibitory concentration
BN: Benin
GH: Ghana
A: Amasaman
N: Nsawam
Az: Azithromycin
Dap: Dapsone
Amk: Amikacin
Cip: Ciprofloxacin
Ofl: Ofloxacin
Clo: Clofazimine.
